# Supplementary material for: Definition of sampling units begets conclusions in ecology: the case of habitats for plant communities
Source: PeerJ. 2015 Mar 5;3:e815. doi: 10.7717/peerj.815 (PMC4358653; doi:10.7717/peerj.815)
Supplement: Table S1 — We used information obtained from bedrock maps (The Geological survey of Norway; www.ngu.no), assigning each target habitat with the correct bedrock type after the field session. [file peerj-03-815-s002.pdf]

Table S1. Number of habitats per district and approach and their corresponding bedrock type. We used information obtained from bedrock maps (The Geological survey of Norway; www.ngu.no), assigning each target habitat with the correct bedrock type after the field session.

				formal approach	subjective approach
			sandstone	3	2
		**east**	sandstone, schist	2	3
			sandstone, schist, calc.	1	1
**mesic habitat**	**district**				
			sandstone	3	2
		**west**	sandstone, schist	0	0
			sandstone, schist, calc.	1	2
			sandstone	1	1
		**east**	sandstone, schist	2	2
			sandstone, schist, calc.	0	0
**snowbed habitat**	**district**				
			sandstone	2	2
		**west**	sandstone, schist	0	1
			sandstone, schist, calc.	2	1
