# Supplementary material for: Definition of sampling units begets conclusions in ecology: the case of habitats for plant communities
Source: PeerJ. 2015 Mar 5;3:e815. doi: 10.7717/peerj.815 (PMC4358653; doi:10.7717/peerj.815)
Supplement: Table S2 — Mixed models contained the approach of defining sampling units, the two districts of different reindeer density and the three bedrock categories as fixed factors. [file peerj-03-815-s003.pdf]

Table S2. Replication of plots according to fixed factors of the mixed models. Mixed models contained the approach of defining sampling units, the two districts of different reindeer density and the three bedrock categories as fixed factors.

				formal approach	subjective approach
**mesic habitat**	**district**	**east**	sandstone	58	34
			sandstone, schist	32	39
			sandstone, schist, calc.	25	16
		**west**	sandstone	46	33
			sandstone, schist	0	0
			sandstone, schist, calc.	16	30
**snowbed habitat**	**district**	**east**	sandstone	21	20
			sandstone, schist	18	24
			sandstone, schist, calc	0	0
		**west**	sandstone	26	27
			sandstone, schist	0	16
			sandstone, schist, calc.	20	16
