# Supplementary material for: Definition of sampling units begets conclusions in ecology: the case of habitats for plant communities
Source: PeerJ. 2015 Mar 5;3:e815. doi: 10.7717/peerj.815 (PMC4358653; doi:10.7717/peerj.815)
Supplement: Table S3 — The table shows Akaike’s Information Criterion (AIC) for each model. “Value” indicates effects of factor levels compared to the Intercept which is followed by a t-test statistic. [file peerj-03-815-s004.pdf]

Table S3. Linear Mixed Effect Models for the mesic habitat type. The table shows Akaike´s Information Criterion (AIC) for each model. “Value” indicates effects of factor levels compared to the Intercept which is followed by a t-test statistic.

Response	Model AIC	Fixed Factors	Value	Std.Error	DF	t-value	p-value
log (bm forbs + 0.7092)	958.69	Intercept - formal approach in eastern district	1.22	0.32	316	3.86	0.000
		sand, schist, calc.	0.29	0.31	316	0.96	0.340
		sand, schist	-1.16	0.31	316	-3.72	0.000
		subjective approach	0.88	0.14	316	6.15	0.000
		western district	-1.15	0.45	7	-2.55	0.038
		subjective approach in western district	-0.44	0.26	316	-1.69	0.092
log (bm prost. *Salix* + 0.7092)	1098.47	Intercept - formal approach in eastern district	0.56	0.35	316	1.57	0.118
		sand, schist, calc.	0.83	0.36	316	2.28	0.023
		sand, schist	-0.53	0.37	316	-1.41	0.161
		subjective approach	0.64	0.18	316	3.57	0.000
		western district	-0.27	0.50	7	-0.55	0.602
		subjective approach in western district	-0.76	0.32	316	-2.39	0.017
log (bm grasses + 0.1258)	1197.04	Intercept - formal approach in eastern district	1.13	0.45	316	2.55	0.011
		sand, schist, calc.	-0.31	0.44	316	-0.70	0.483
		sand, schist	-1.26	0.45	316	-2.81	0.005
		subjective approach	0.83	0.21	316	4.00	0.000
		western district	-0.90	0.64	7	-1.41	0.202
		subjective approach in western district	-0.73	0.37	316	-1.96	0.051
log (bm grasses silica + 0.1258)	1112.86	Intercept - formal approach in eastern district	-0.31	0.31	316	-1.01	0.312
		sand, schist, calc.	-0.40	0.34	316	-1.19	0.236
		sand, schist	-0.51	0.36	316	-1.43	0.154
		subjective approach	-1.39	0.18	316	-7.62	0.000
		western district	-1.58	0.43	7	-3.66	0.008
		subjective approach in western district	1.79	0.32	316	5.60	0.000
log (bm dec. woody + 0.7092)	806.85	Intercept - formal approach in eastern district	0.10	0.12	316	0.77	0.441
		sand, schist, calc.	0.06	0.15	316	0.42	0.672
		sand, schist	-0.58	0.17	316	-3.51	0.001
		subjective approach	0.23	0.11	316	2.01	0.046
		western district	-0.28	0.17	7	-1.62	0.149
		subjective approach in western district	-0.37	0.19	316	-1.98	0.049
log (bm evergreens + 0.2704)	1106.36	Intercept - formal approach in eastern district	-0.19	0.25	316	-0.79	0.432
		sand, schist, calc.	-0.02	0.28	316	-0.06	0.955
		sand, schist	-0.33	0.31	316	-1.05	0.297
		subjective approach	0.17	0.18	316	0.93	0.352
		western district	-0.85	0.34	7	-2.49	0.042
		subjective approach in western district	0.39	0.31	316	1.27	0.205
log (bm *Betula n.*+ 1.4706)	1051.02	Intercept - formal approach in eastern district	1.59	0.34	314	4.72	0.000
		sand, schist, calc.	-0.32	0.34	314	-0.95	0.345
		sand, schist	0.13	0.38	314	0.35	0.729

		subjective approach	-0.70	0.18	314	-3.92	0.000
		western district	-0.91	0.49	7	-1.88	0.102
		subjective approach in western district	1.09	0.30	314	3.61	0.000
log (bm *Vaccinium m.*+ 0.7092)	1252.04	Intercept - formal approach in eastern district	1.12	0.70	316	1.59	0.112
		sand, schist, calc.	-1.01	0.54	316	-1.89	0.060
		sand, schist	2.63	0.53	316	5.00	0.000
		subjective approach	-0.78	0.22	316	-3.45	0.001
		western district	1.15	1.02	7	1.13	0.296
		subjective approach in western district	0.35	0.42	316	0.83	0.406
log (bm *Empetrum h.*+ 1.5804)	1295.20	Intercept - formal approach in eastern district	1.86	0.71	316	2.61	0.010
		sand, schist, calc.	-0.86	0.57	316	-1.51	0.131
		sand, schist	2.68	0.56	316	4.80	0.000
		subjective approach	-0.57	0.24	316	-2.39	0.018
		western district	0.84	1.04	7	0.81	0.443
		subjective approach in western district	0.12	0.44	316	0.27	0.790
Gini-Simpson Index	-169.07	Intercept - formal approach in eastern district	0.60	0.05	314	12.40	0.000
		sand, schist, calc.	-0.01	0.05	314	-0.16	0.872
		sand, schist	-0.11	0.06	314	-1.93	0.055
		subjective approach	0.03	0.03	314	1.02	0.308
		western district	-0.16	0.07	7	-2.22	0.062
		subjective approach in western district	0.07	0.05	314	1.51	0.133
Shannon Index	368.23	Intercept - formal approach in eastern district	1.41	0.17	316	8.29	0.000
		sand, schist, calc.	-0.05	0.13	316	-0.39	0.699
		sand, schist	-0.83	0.13	316	-6.23	0.000
		subjective approach	0.18	0.06	316	3.09	0.002
		western district	-0.57	0.25	7	-2.30	0.055
		subjective approach in western district	0.09	0.11	316	0.88	0.378
Species Richness	1579.33	Intercept - formal approach in eastern district	7.81	1.24	314	6.30	0.000
		sand, schist, calc.	-0.39	0.91	314	-0.43	0.668
		sand, schist	-3.26	1.02	314	-3.20	0.002
		subjective approach	3.24	0.42	314	7.74	0.000
		western district	-2.41	1.97	9	-1.22	0.253
		subjective approach in western district	-0.96	0.72	314	-1.34	0.180
