# Supplementary material for: Definition of sampling units begets conclusions in ecology: the case of habitats for plant communities
Source: PeerJ. 2015 Mar 5;3:e815. doi: 10.7717/peerj.815 (PMC4358653; doi:10.7717/peerj.815)
Supplement: Table S4 — The table shows Akaike’s Information Criterion (AIC) for each model. “Value” indicates effects of factor levels compared to the Intercept which is followed by a t-test statistic. [file peerj-03-815-s005.pdf]

Table S4. Linear Mixed Effect Models for the snowbed habitat type. The table shows Akaike´s Information Criterion (AIC) for each model. "Value" indicates effects of factor levels compared to the Intercept which is followed by a t-test statistic.

Response	Model AIC	Fixed Factors	Value	Std.Error	DF	t-value	p-value
log (bm forbs + 0.1388)	679.46	Intercept - formal approach in eastern district	0.61	2.33	178	0.26	0.794
		sand, schist, calc.	-6.25	0.82	178	-7.60	0.000
		sand, schist	-3.43	0.44	178	-7.73	0.000
		subjective approach	2.73	0.33	178	8.31	0.000
		western district	2.54	2.86	4	0.89	0.425
		subjective approach in western district	-3.15	0.45	178	-7.07	0.000
log (bm prost. *Salix* + 0.7092)	667.86	Intercept - formal approach in eastern district	3.07	0.25	176	12.49	0.000
		sand, schist, calc.	0.27	0.29	176	0.93	0.355
		sand, schist	-0.40	0.24	176	-1.66	0.099
		subjective approach	-1.01	0.30	176	-3.35	0.001
		western district	-2.14	0.34	6	-6.40	0.001
		subjective approach in western district	1.82	0.41	176	4.47	0.000
log (bm grasses + 0.1258)	690.53	Intercept - formal approach in eastern district	1.43	0.85	178	1.68	0.094
		sand, schist, calc.	-0.10	0.74	178	-0.13	0.895
		sand, schist	-0.96	0.43	178	-2.21	0.028
		subjective approach	0.88	0.34	178	2.57	0.011
		western district	-0.66	1.06	4	-0.63	0.563
		subjective approach in western district	-0.93	0.46	178	-2.03	0.044
log (bm grasses silica + 0.1258)	578.58	Intercept - formal approach in eastern district	-0.81	0.24	176	-3.38	0.001
		sand, schist, calc.	-0.19	0.33	176	-0.57	0.568
		sand, schist	0.10	0.24	176	0.43	0.670
		subjective approach	-1.16	0.26	176	-4.46	0.000
		western district	-0.82	0.33	6	-2.45	0.050
		subjective approach in western district	0.76	0.34	176	2.25	0.026
log (bm Sedges + 0.2324)	617.52	Intercept - formal approach in eastern district	-0.22	0.43	178	-0.52	0.601
		sand, schist, calc.	-0.10	0.48	178	-0.21	0.831
		sand, schist	0.56	0.32	178	1.77	0.079
		subjective approach	0.01	0.28	178	0.02	0.982
		western district	-0.35	0.54	4	-0.65	0.553
		subjective approach in western district	-0.42	0.37	178	-1.14	0.256
log (bm *Vaccinium m.*+ 0.7092)	494.60	Intercept - formal approach in eastern district	-0.02	0.50	176	-0.05	0.961
		sand, schist, calc.	0.51	0.48	176	1.06	0.291
		sand, schist	-0.13	0.34	176	-0.38	0.706
		subjective approach	0.03	0.28	176	0.09	0.928
		western district	0.05	0.66	4	0.08	0.942
		subjective approach in western district	0.11	0.33	176	0.34	0.737
log (bm *Empetrum h.*+ 1.5804)	265.37	Intercept - formal approach in eastern district	0.36	0.15	178	2.35	0.020
		sand, schist, calc.	0.21	0.18	178	1.16	0.249
		sand, schist	0.26	0.12	178	2.22	0.028
		subjective approach	0.02	0.10	178	0.23	0.817
		western district	0.11	0.20	4	0.58	0.596
		subjective approach in western district	-0.06	0.14	178	-0.45	0.650
Gini-Simpson Index	1.60	Intercept - formal approach in eastern district	0.50	0.07	176	7.06	0.000
		sand, schist, calc.	-0.24	0.10	176	-2.42	0.017
		sand, schist	-0.02	0.07	176	-0.24	0.812

		subjective approach	0.05	0.06	176	0.72	0.475
		western district	0.09	0.10	4	0.94	0.399
		subjective approach in western district	-0.13	0.08	176	-1.71	0.089
Shannon Index	268.49	Intercept - formal approach in eastern district	1.14	0.47	178	2.44	0.016
		sand, schist, calc.	-1.42	0.26	178	-5.47	0.000
		sand, schist	-0.59	0.14	178	-4.18	0.000
		subjective approach	0.51	0.11	178	4.81	0.000
		western district	0.52	0.58	4	0.90	0.419
		subjective approach in western district	-0.75	0.14	178	-5.19	0.000
Species Richness	916.34	Intercept - formal approach in eastern district	8.26	4.51	178	1.83	0.069
		sand, schist, calc.	-13.95	1.58	178	-8.84	0.000
		sand, schist	-6.06	0.85	178	-7.12	0.000
		subjective approach	4.73	0.63	178	7.51	0.000
		western district	4.64	5.54	4	0.84	0.449
		subjective approach in western district	-6.16	0.86	178	-7.20	0.000
